# Dual action tofacitinib-loaded PLGA nanoparticles alleviate colitis in an IBD mouse model

**DOI:** 10.1007/s13346-024-01736-1

**Published:** 2024-11-11

**Authors:** Nidhi Seegobin, Laura E. McCoubrey, Cécile Vignal, Christophe Waxin, Youssef Abdalla, Yue Fan, Atheer Awad, Sudaxshina Murdan, Abdul W. Basit

**Affiliations:** 1 Department of Pharmaceutics, UCL School of Pharmacy, University College London, WC1N 1AX 29-39 Brunswick Square, London, UK; 2 Univ. Lille, Inserm, CHU Lille, UMR1286 - INFINITE - Institute for Translational Research in Inflammation, 59000 Lille, France; 3 Drug Product Development, GSK R&D, Ware, SG12 0GX UK; 4 Department of Clinical, Pharmaceutical and Biological Sciences, University of Hertfordshire, College Lane, Hatfield, AL10 9AB UK

**Keywords:** Inflammatory bowel disease, PLGA, Microbiome medicine, Nanoparticles, Tofacitinib, Nanoprecipitation, Murine model of acute colitis, Colonic drug delivery and targeting

## Abstract

Inflammatory bowel disease (IBD) affects over 7 million people worldwide and significant side effects are associated with current therapies such as tofacitinib citrate (TFC), which is linked to increased risks of malignancy and congestive heart issues. To mitigate these systemic adverse effects, localised drug delivery via nano-sized carriers to inflamed gut tissues represents a promising approach. Herein, we aimed to optimise the synthesis of nanoparticles (NPs) using a low molecular weight grade of Poly(lactic-co-glycolic acid) (PLGA) 50:50 loaded with TFC. This approach leverages the dual anti-inflammatory action of TFC and the local production of anti-inflammatory short-chain fatty acids from the degradation of PLGA by colonic gut microbiota. NPs were produced by nanoprecipitation and characterised for their drug release profile in vitro. The efficacy of the enhanced PLGA-TFC NPs was then tested in a C57BL/6 DSS colitis mouse model. The release profile of TFC from the enhanced PLGA NPs showed a 40% burst release within the first hour, followed by up to 80% drug release in the colonic environment. Notably, the degradation of PLGA by colonic gut microbiota did not significantly influence TFC release. In the mouse model, neither PLGA NPs alone nor TFC alone showed significant effects on weight loss compared to the TFC-loaded PLGA NPs, emphasising the enhanced efficacy potential of the combined formulation. Altogether, these results suggest a promising role of NP delivery systems in enhancing TFC efficacy, marking a significant step towards reducing dosage and associated side effects in IBD treatment. This study underscores the potential of PLGA-TFC NPs in providing targeted and effective therapy for IBD.

**Graphical Abstract:**

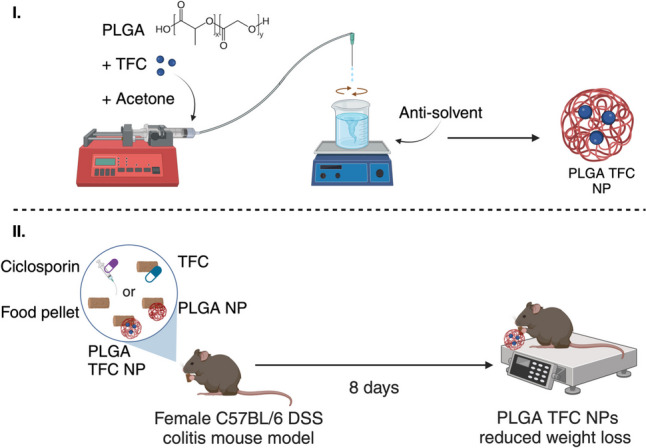

**Supplementary Information:**

The online version contains supplementary material available at 10.1007/s13346-024-01736-1.

## Introduction

Inflammatory bowel disease (IBD) is an umbrella term used to describe two inflammatory conditions of the gastrointestinal (GI) tract, ulcerative colitis (UC) and Crohn’s disease (CD) affecting over 7 million individuals globally [1]. The limited efficacy of current IBD treatments, often accompanied by significant side effect profiles, has prompted the exploration of novel therapeutic modalities [2, 3]. In recent years, this pursuit has led to the emergence of novel anti-inflammatory drugs, such as the Janus kinase (JAK) inhibitors. JAK inhibitors modulate intracellular signal transductions critical to the progression of immune and inflammatory responses thereby preventing the release of inflammatory proteins including over 50 soluble factors [4, 5]. This mechanism of action contrasts significantly with existing therapies on the market, which primarily target specific extracellular cytokines, such as TNF-α inhibitors. However, the clinical application of these therapies has been marred by significant side effects, including an elevated risk of malignancies, cardiovascular disorders, and venous thromboembolism [6, 7]. Tofacitinib (TFC), a recently marketed JAK inhibitors for the treatment of mild to severe UC, has garnered attention due to its safety concerns, leading to regulatory interventions such as an United States Food and Drug Administration (FDA) black box warning and a Medicines and Healthcare products Regulatory Agency (MHRA) black triangle warning [8, 9]. Current usage guidelines advise against its administration in specific patient groups, such as those aged 65 years or older, individuals with a history of long-term smoking, and patients with cardiovascular disease or malignancy risk factors, unless no suitable alternatives are available [9]. Reducing adverse effects associated with JAK inhibitors could serve as a strategic approach for the cautious reintroduction of these drugs to a broader patient demographic. The current marketed dosage form of TFC is available as both a twice-daily immediate release tablets and a once-daily modified release tablets ensuring more convenient dosing for patients [6, 10, 11], however no colonic targeted formulations are available.

Local drug delivery of UC treatments via colonic targeting aims to reduce systemic side effects by enabling high drug concentrations at the site of inflammation [12, 13]. This approach bypasses absorption in the upper GI tract and minimises systemic drug exposure, which typically occurs within one hour of administration with immediate release formulations [14]. Various strategies have been demonstrated to achieve colonic drug delivery, including colon-targeted formulations and azo-bonded prodrugs of TFC [15, 16]. Work by Yadav et al. [16] was particularly promising, they showed that ileocolonic-targeted TFC capsules decreased systemic drug exposure, increased colonic tissue exposure and reduced the levels of the pro-inflammatory cytokine IL-6 in an LPS-induced acute rat model of inflammation [16]. Altogether these results suggest that a reduction in side effects by local drug delivery is possible. As such, a drug delivery system specifically designed to minimise systemic side effects associated with TFC while enhancing its local tissue concentrations, is highly needed [17]. One approach to reduce TFC-associated systemic side effects by is through inflammation-targeted colonic drug delivery. This method may enable more precise delivery to the inflamed tissue, characterised by the “leaky gut” phenomenon due to gaps in the tight junctions of the epithelial cells. Such a system could employ negatively charged synthetic nanoparticles (NPs) to pass through the leaky gut and target the positively charged inflamed areas [18–20]. In this study, we aim to load TFC into poly(lactic-co-glycolic acid) (PLGA) NPs to achieve this targeted delivery [21–23].

PLGA is a biocompatible and biodegradable polymer composed of repeating units of lactic acid and glycolic acid monomers. PLGA’s versatility in drug delivery arises from its ability to be tailored with different ratios of lactic acid (LA) to glycolic acid (GA), which influence its hydrolytic degradation kinetics [24]. In vivo, the ester bonds in PLGA are hydrolysed into lactic acid and glycolic acid, which are naturally occurring and exit the body by Krebs cycle metabolism as carbon dioxide and water [25]. This unique feature makes PLGA an attractive choice for controlled drug release applications, and it has been approved for pharmaceutical applications by the FDA and the European Medicines Agency (EMA), one of the most notable marketed products being the Zoladex Depot® [26]. PLGA has been extensively used for the formulation of NPs and investigated for the oral drug delivery of several compounds [27–30]. Apart from its established role as a drug carrier, PLGA has recently gained attention for its own impact on the gut microbiome and colonic health. Recently, it has been shown that a low molecular weight (MW) grade (2000–2500 g/mol) of PLGA is metabolised into lactate which is a precursor in the production of short-chain fatty acids (SCFAs) by the human colonic microbiota and found to reduce the expression of inflammatory markers (Interleukin(IL)-8 and IL-10) in a colonic in vitro model of inflammation [31]. Indeed, SCFAs (i.e. propionate and butyrate) mainly originate from bacterial fermentation of dietary fibre in the colon and are involved in maintaining epithelial barrier function, suppressing colitis, and protecting against immune disorders [32–34]. While PLGA is susceptible to hydrolysis, a studies have shown that PEGylated or hyaluronic acid functionalised PLGA nanoparticles can maintain stability within the upper GI environment for colonic targeting [35, 36]. Given the potential benefits of encapsulating TFC to target inflammation in the inflamed leaky gut and of its local colonic delivery (to reduce systemic side effects), we hypothesised that encapsulating TFC within low molecular weight PLGA nanoparticles would be a promising dual-action strategy to treat colitis. TFC’s role would be to reduce inflammation, while PLGA would form the nanoparticle carrier and its metabolism in the colon would result in the formation of the beneficial SCFAs. To test this hypothesis, PLGA particles loaded with TFC were produced, characterised in vitro, and then evaluated in a C57BL/6 DSS colitis mouse model. In this paper, we report on the preparation and physico-chemical characterisation of blank and of drug-loaded PLGA particles, and on their in vivo action in a colitis mouse model.

## Materials and methods

### Materials

Resomer® Condensate RG 50:50 MN 2300 (PLGA, acid terminated, 50:50, Mw 2000 – 2500 g/mol) AND Resomer® R 202 H, (PLA, acid terminated Mw 10,000—18,000 g/mol) were purchased from Evonik Industries (Essen, Germany). Acetone, Bryant and Burkey broth medium, lipase from porcine pancreas, glycolic acid, poloxamer 407 (Kolliphor® P 407), potassium phosphate monobasic, hexadecyltrimethylammonium bromide, dianisidine dihydrochloride and human neutrophil MPO were purchased from Merck Life Science (Gillingham, UK). Anaerogen packs, purified pepsin (derived from porcine stomach mucosa with an activity of 2,000—2,400 units/mg), sodium chloride, phosphoric acid, HPLC-grade acetonitrile, lecithin, N, N-Dimethylacetamide (DMA), Dextran Sulphate Sodium (DSS) and SYBR Green PCR master mix were purchased from Fisher Scientific (Loughborough, UK). Sodium taurocholate and TFC (MW: 504.49 g/mol) were purchased from Cambridge Bioscience Ltd. (Cambridge, UK). Sodium hydroxide pellets were purchased from VWR International (Pennsylvania, USA). Hydrochloric acid was purchased from LP Chemicals Ltd. (Winsford, UK). Nucleospin RNA II kit was purchased from Macherey–Nagel (Hoerdt, France). Where used, water was of HPLC-grade and obtained via an ELGA HPLC water purification system (ELGA LabWater, High Wycombe, UK).

### Preparation of TFC-loaded PLGA NPs by nanoprecipitation

**Fig. 1:**
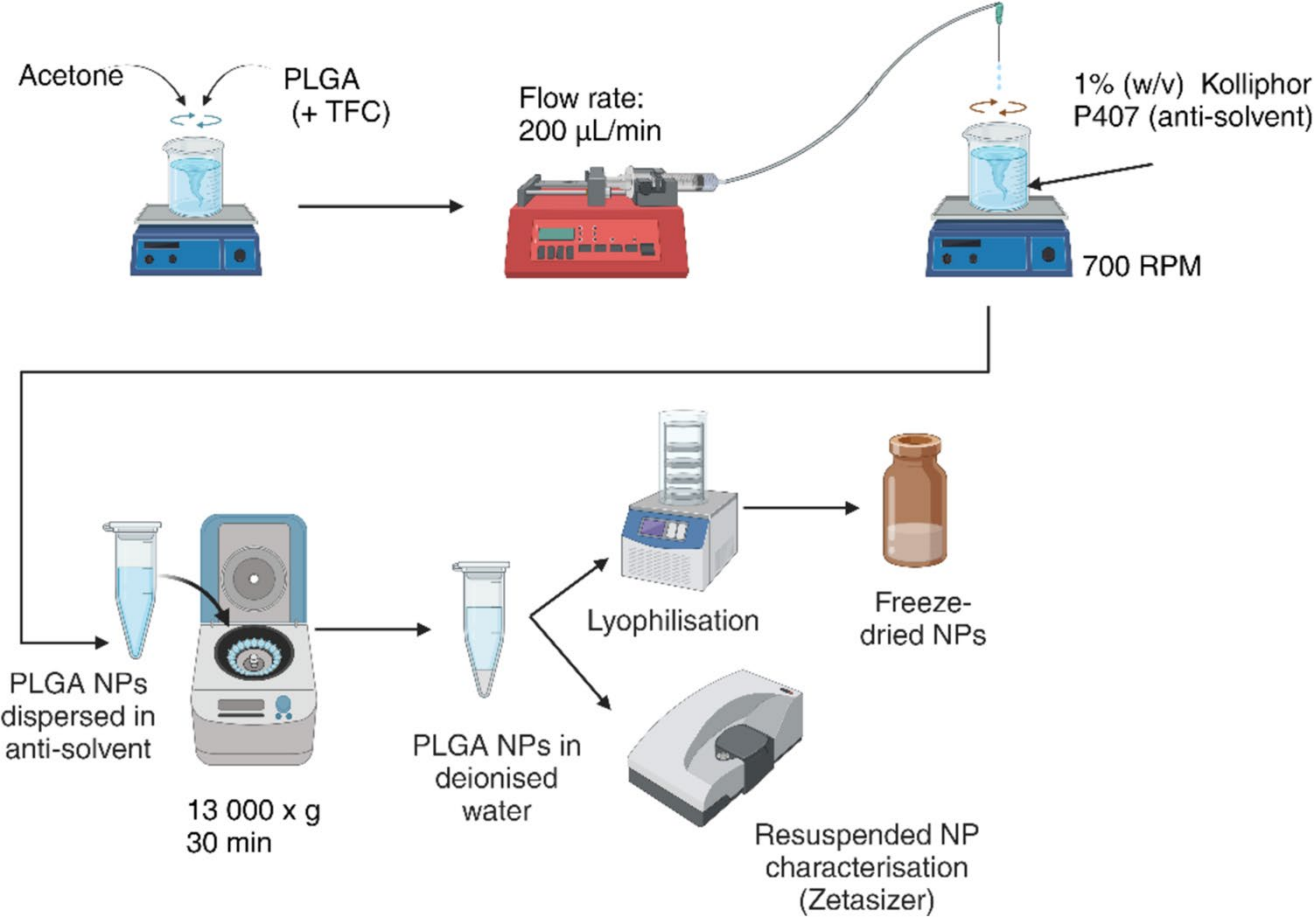
Schematic representation of the nanoprecipitation process used for the production of the PLGA TFC NPs, where a mixture of polymer (± drug) and acetone was added dropwise into an aqueous medium containing 1% (w/v) poloxamer 407

PLGA NPs were prepared in triplicate using the nanoprecipitation method (Fig. 1). PLGA and TFC were dissolved in acetone using a range of concentration combinations based on a full factorial design of experiment JMP® (SAS institute, United Kingdom), (Supplementary Table S1). TFC was first dissolved in acetone with the aid of brief sonication prior to the addition and dissolution of PLGA into the drug- acetone solution. The preparation method is schematically shown in Fig. 1. An AL-1000 syringe driver (Precision Instruments, Hitchin, UK) at 200 µL/min was attached to a 30 G × 0.5″ needle (Microlance™ 3, Becton Dickinson, New Jersey, USA). This system was used to dispense droplets of polymer-drug organic solutions into an anti-solvent solution of 1% (w/v) poloxamer 407 in deionised water. The anti-solvent liquid was stirred throughout with a magnetic bar rotating at 700 rpm. The final ratio of polymer (± drug) solution to anti-solvent liquid after droplet precipitation was 1:2 v/v. Acetone was then allowed to evaporate from the uncovered mixture overnight by stirring at 700 rpm at room temperature (25 °C). The resultant nanosuspension was centrifuged (3-16KL Centrifuge, Sigma Laborzentrifugen, Osterode am Harz, Germany) at 10,500xg, for 30 min at 4 °C. After centrifugation the supernatant was discarded, and the NPs were resuspended in 1.5 mL cool (2—8 °C) deionised water, in order to remove any unentrapped TFC and to maintain the polymer-based particle in a rigid state, below its glass transition temperature of around 30 °C [37]. The NPs were subsequently sized via dynamic light scattering (DLS) and frozen at -80 °C in glass vials immediately prior to lyophilisation. A BUCHI™ Lyovapor™ L-300 (BUCHI™, Flawil, Switzerland) was used to freeze-dry the samples, with a drying temperature of -80 °C and a pressure of 0.50 mbar. Upon the completion of the process, the lyophilised samples were returned to atmospheric pressure and stored at -20 °C until further analysis.

### NP characterisation

#### Particle size and stability by DLS

The NP resuspension was analysed for size, polydispersity index (PDI) and zeta (ζ) potential prior to lyophilisation. This was done using a Malvern ZetaSizer (Malvern Panalytical Ltd., Malvern, UK). Measurements (*n* = 3) were conducted at 25 °C with an equilibration time of 120 s. The measurement settings were 173° backscatter (NIBS default), with an automatic measurement duration.

#### Drug loading

The drug entrapment efficiency of the NP formulations was calculated by an indirect method which involved sedimentation of NPs by centrifugation followed by analysis of free drug in the supernatant. The difference between the amount of drug added initially in the formulation and free drug found in the supernatant was calculated as the amount of drug entrapped in the formulation. The NP suspensions were centrifuged at 10,000 × g for 30 min, which caused the NPs to sediment. Subsequently, the supernatant was analysed using ultraviolet–visible light (UV–Vis) spectrophotometry (Cary 100, Agilent, USA) at 286 nm after a tenfold dilution with deionised water. The % entrapment efficiency of the formulation was calculated as follows (Eq. 1):

1 $$\%\; Entrapment=\frac{Drug\; in\; formulation -Drug\; in\; supernatent}{Drug\; in\; formulation} \times 100$$

The drug loading capacity was calculated as the percentage of drug mass per mass of drug-loaded polymeric particles (Eq. 2). Briefly, about 5 mg of dried NPs were dissolved in DMA which was then diluted tenfold and analysed for drug content using UV–Vis spectrophotometry (Cary 100, Agilent, USA) at 286 nm. Drug content was calculated using a calibration curve.

2 $$\%\; Drug\; loading=\frac{Mass\; of\; drug\; in\; particle }{Mass\; of\; drug\; and\; polymer}\times 100$$

#### Product and drug yields

The product yield was calculated by weighing the total amount of dried NPs following lyophilisation, as per Eq. 3. The TFC yield was obtained as per Eq. 4, by calculating the total amount of TFC in the dried NPs based on the drug loading capacity assay (Section "Estimating polymer degradation in simulated GI fluids") in combination with the total amount of dried NPs.

3 $$\%\; Product\; yield=\frac{mass\; of\; dried\; nanoparticles}{initial\; drug\; and\; polymer\; mass}\times 100$$

4 $$\%\; TFC\; yield=\frac{mass\; of\; TFC\; in\; dried\; nanoparticles}{initial\; total\; drug\; mass}\times 100$$

#### Selection of the enhanced NP formulation

The enhanced formulation of TFC PLGA NPs for use in the animal study was determined through a comprehensive assessment integrating multiple parameters, including particle size, stability, drug entrapment efficiency, and yield of TFC. The selection of a suitable particle size range, typically between 20 and 150 nm, was based on its potential to exploit the leaky gut and enhanced permeation and retention (EPR) effect, facilitating preferential accumulation within inflamed tissues while sparing healthy colonic tissues [22, 23, 38, 39]. Furthermore, stability, a critical attribute of NP formulations, was evaluated using ζ potential measurements, with a threshold of ± 30 mV indicating satisfactory stability [40, 41]. Finally, the formulation’s drug loading capacity and overall yield were considered in the optimisation process.

#### Scanning Electron Microscopy of enhanced NP formulation

Scanning electron microscopy (SEM) was employed to analyse the morphology of PLGA particles. A small amount of the liquid sample was uniformly spread onto a 25 mm aluminium stub using self-adhesive carbon tape (Taab Laboratory Equipment, Reading, UK). Subsequently, a thin layer of gold was sputtered onto the sample surface for 60 s at a current of 20 mA using the Quorum Q150R Plus Rotary Pumped Coater (Quorum, Laughton, UK). Examination of the particles was performed using the Phenom Pro Desktop SEM (Fisher Scientific, Loughborough, UK) at an accelerating voltage of 10 kV. Freeze-dried particles underwent examination with a Quanta 200 FEG SEM (FEI Company, Oregon, USA) operating at an accelerating voltage of 50 kV. Digital images were captured and analysed.

#### Preparation of simulated GI fluids

##### FaSSGF and FaSSIF

The fluids used to investigate the particles’ GI stabilities were biorelevant fasted state simulated gastric fluid (FaSSGF) (pH 1.6) and fasted state simulated intestinal fluid (FaSSIF) (pH 6.5), both supplemented with lipase, sodium taurocholate, and lecithin. Lipase was added to the fluids in biorelevant concentrations as it has been shown to digest PLA and PLGA [42, 43]. Supplementary Table S2 shows the composition of the simulated fluids used, which were prepared as previously described [44].

##### Culture of colonic bacteria

Frozen colonic microbiotas were obtained from the faeces of six healthy adult human donors (3 males and 3 females) with an average of 9.03 × 10^8^ ± 1.51 × 10^8^ CFU/mL as described in the Supplementary Section S3. This approach was taken to account for individual diversity in gut microbiota, thereby enhancing the accuracy of the study [45, 46]. The colonic microbiota samples were thawed for 1 min at 37 °C and pooled together. Two mL of the pooled mixture was used to inoculate 200 mL of Bryant and Burkey (BB) broth under anaerobic conditions (A20 Sleeved Anaerobic Workstation, Don Whitley Scientific, Bingley, UK; containing 5% CO_2_, 5% H_2_, 90% nitrogen, set at 37 °C). The bacterial broth was left to incubate for 16 h at 37 °C, prior to its use in the drug release study. At the 16-h mark, the broth was plated onto BB agar plates, and the colonies were counted after 96 h of incubation under anaerobic conditions. This was conducted to ensure a bacterial concentration reflective of the human colonic environment, typically exceeding 10^10–11^ CFU/mL [47–49].

### PLGA particle stability in the GI fluids

To assess the stability of PLGA NPs in simulated GI fluids, two sizes of PLGA particles (samples A and B) were prepared using two different concentrations PLGA 10 mg/mL and 50 mg/mL according to the methods described in Section "Preparation of TFC-loaded PLGA NPs by nanoprecipitation". Drug-free polylactic acid (PLA) particles (using PLA at 10 mg/mL) were also prepared by nanoprecipitation as a control.

#### Incubation of PLGA particles in simulated GI fluids

Biorelevant FaSSGF and FaSSIF were prepared the day before incubation and pre-warmed to 37 °C in an incubator shaker (Innova 4000, New Brunswick Scientific, New Jersey, USA). The pH of the fluids was measured using a calibrated pH meter with automatic temperature compensation (Hi-2020 Edge Hybrid Multiparameter, Hanna Instruments, Rhode Island, USA). Then, FaSSGF and FaSSIF were added to blank PLGA particles in glass vials to achieve a polymer concentration of 40 mg/mL. This concentration was selected to facilitate detection of acid release following polymer degradation, if any, and to be well below the maximum solubility of lactate (100 mg/mL) thus providing sink conditions.

Each PLGA particle-FaSSGF and PLGA particle-FaSSIF combination was incubated in triplicate in glass vials maintained at 37 °C and shaken at 150 rpm for 1.5 h (FaSSGF) or 4.5 h (FaSSIF). These timeframes were chosen to reflect the upper range of gastric and small intestinal transit times, respectively [50]. At the end of the incubation period the pH of the fluids was measured, whereby a statistically significant decrease in pH was taken as an indication of PLGA degradation and release of acidic monomers, lactic acid and glycolic acid.

One size of PLA NPs (146.3 ± 0.59 nm) was used as a positive control, based on the recent work that demonstrated PLA digestion by mammalian lipase in simulated gastric and small intestinal fluid [42]. The same concentrations of lipase as in reference [42] were used to facilitate comparison of results. A second control was incubation of FaSSGF/FaSSIF in the absence of polymeric particle, to verify that any pH change observed in the polymeric particle incubations was due to polymer degradation, rather than pH change in the simulated fluids over time. A third control was incubation of PLGA A (10 mg/mL) nanoparticles (144.5 ± 1.31 nm) and PLGA B (50 mg/mL) nanoparticles (313.2 ± 11.81 nm) in a blank FaSSIF, i.e. one without lipase, sodium taurocholate, and lecithin to determine polymer stability in such environments.

#### Estimating polymer degradation in simulated GI fluids

The expected pH resulting from 5% (w/v) PLA degradation in FaSSGF which has no buffer capacity was calculated using Eq. 5 which depicts the hydrogen ion concentration (mol/L) in a fluid with a known pH measured with temperature compensation.

5 $$\left[{H}^{+}\right]={10}^{-pH}$$

The amount of PLGA degradation in FaSSIF and blank FaSSIF was approximated by measuring the pH changes in these media in response to addition of lactic acid and glycolic acid. FaSSIF and blank FaSSIF were prepared on the morning of experiment. Then, ascending concentrations of lactate and glycolate, in equal molar ratios, were added to 100 mL of the fluids under stirring (Table 1). These concentrations were selected to reflect the concentrations of lactate and glycolate that would be released from PLGA during a 40 mg/mL polymer incubation. After each stage of lactate and glycolate addition, the pH of the fluids was measured with the calibrated pH meter. Measurements were conducted in triplicate for both types of FaSSIF. In this manner, the percentage of PLGA degraded during the FaSSIF incubations could be approximated, based on the pH changes recorded after 4.5 h.

**Table 1:** The effect of ascending concentrations of lactate and glycolate on the pH of blank FaSSIF and FaSSIF. Lactate and glycolate concentrations were selected to correspond to specific percentages of PLGA degradation when PLGA NP was incubated at 40 mg PLGA/mL in the simulated fluids. Measurements were conducted in triplicate for each fluid and are shown as averages ± standard deviations

PLGA degradation (%)	Concentration PLGA degraded (mg/mL)	Concentration lactate released (mg/mL)	Concentration glycolate released (mg/mL)	pH blank FaSSIF	pH FaSSIF
0	0.00	0.00	0.00	6.49 ± 0.01	6.47 ± 0.00
1	0.40	0.216	0.184	6.20 ± 0.02	6.19 ± 0.02
2	0.80	0.432	0.368	5.70 ± 0.02	5.71 ± 0.03
2.5	1.00	0.540	0.460	-	5.23 ± 0.08
3	1.20	0.648	0.552	-	4.60 ± 0.06
3.5	1.40	0.756	0.644	-	4.24 ± 0.05

The buffer capacity of the fluids in response to PLGA degradation was calculated using Eq. 6, whereby the pH changes equivalent to 2% PLGA degradation were used.

6 $$B=\frac{n}{\Delta pH}$$

With *n* = the number of moles of lactate and glycolate added per L of simulated fluid. ΔpH = the average difference in fluid pH before and after addition of 4.8 mM lactate and glycolate (9.6 mM combined, equivalent to 2% PLGA degradation in Table 1).

### *In vitro* TFC release from PLGA nanoparticles exposed to intestinal conditions

**Fig. 2:**
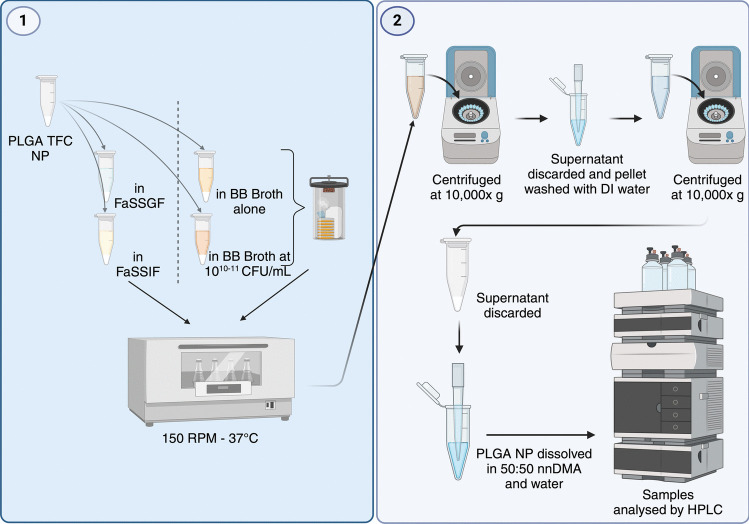
Experimental methods for the measurement of TFC-release from PLGA nanoparticles in simulated GI fluids (*n* = 3)

For measurement of drug release from PLGA nanoparticles, the enhanced NPs produced using 10 mg/mL PLGA and 0.5 mg/mL TFC formulation were exposed to simulated intestinal fluids for 1.5 h in simulated gastric fluid, 4.5 h in simulated intestinal fluids and 24 h in simulated colonic fluids, after which the drug remaining in the nanoparticle was measured (Fig. 2). PLGA TFC NPs were placed in Eppendorf tubes and FaSSGF, FaSSIF or BB broth were added to achieve a polymer concentration of 10 mg/mL. This concentration was chosen to facilitate detection of TFC release from the polymers and to be well below the solubility of TFC (2.9 mg/mL) [51] thus providing sink conditions. The samples were placed in an incubator shaker set at 150 rpm and 37 °C (Innova 4000, New Brunswick Scientific, New Jersey, USA) with the colonic bacterial cultures maintained under anaerobic conditions via the use of AnaeroGen packs. For each timepoint, samples (*n* = 3) were centrifuged at 10,000 × g, the pellets were resuspended with deionised water and centrifuged again before dissolving the pellet in 50:50 DMA and water [52] for analysis using High-performance liquid chromatography (HPLC; Hewlett Packard 1260II Series HPLC system, Agilent Technologies, Cheadle, UK). An isocratic mobile phase comprising 50% water, and 50% methanol was utilised. A 20 µL injection volume was introduced into a Rastak C18 column (150 × 4.6 mm, 5 μm) in reverse phase mode, with a flow rate of 1.0 mL/min. The detection was set at 254 nm, and temperature control was not applied during the process.

#### Evaluation of the efficacy of PLGA and PLGA-TFC nanoparticles in a DSS-induced colitis mouse model

**Fig. 3:**
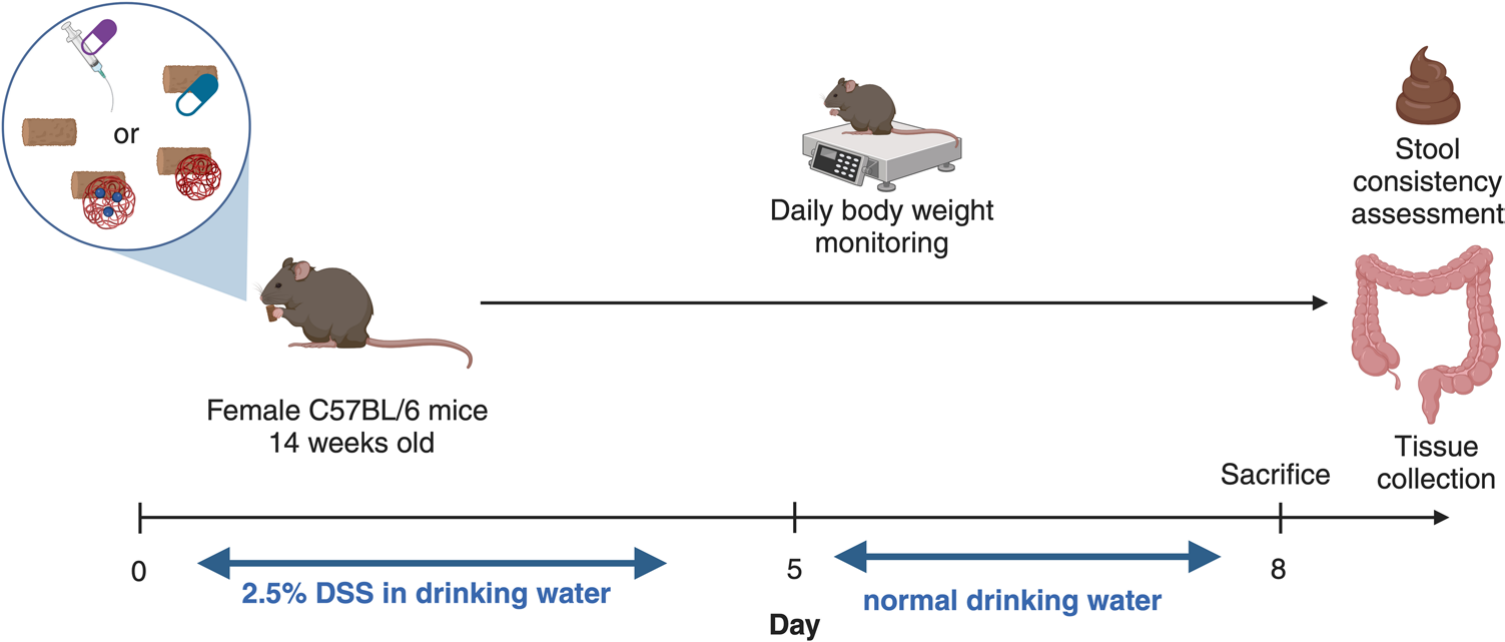
Schematic representation of the in vivo experimental set-up for the therapeutic assay of (i) PLGA NPs, (ii) TFC PLGA NPs or (iii) TFC in DSS-induced colitis C57BL/6 mice (*n* = 8) with a positive control (ciclosporin) and a negative control (usual food pellet diet)

Mice experiments were performed in accordance with the approval (Authorisation No. 7267–2016100410284439) from the Ethics Committee in Animal Experimentation Nord-Pas de Calais (CEEA75) which was approved by the French ministry for higher education and research. The CEEA75 carries out its activity according to the principles of European directive 2010/63/EU (transposed into French law 2013/2/1/AGRG1238767A) and the “National Charter on the ethics of animal experimentation” while respecting the recommendations for specific procedures issued by the National Ethics Reflection Committee on animal experimentation. At the outset of the study, female mice were selected to control for potential sex-related differences in physiological responses. Female C57BL/6 mice aged 14 weeks (Janvier Labs, Le Genest-Saint-Isle, France) were housed under standard conditions. As represented in Fig. 3, eight mice were used per group and administered 2.5% (w/v) Dextran Sulphate Sodium (DSS) in their drinking water over the course of 5 days to induce colitis. The DSS mouse model was chosen due to its ability to replicate pathologies similar to ulcerative colitis, primarily by damaging colonic epithelial cells and compromising the mucosal barrier. This was particularly relevant, as the PLGA condensate used in this study was found to enhance the production of anti-inflammatory short-chain fatty acids through digestion by the colonic gut microbiota. For 5 consecutive days, mice received either (i) PLGA NPs (2.2 mg PLGA/day/mouse), or (ii) PLGA TFC NPs (2.2 mg PLGA/day/mouse, 0.26 mg TFC/kg/day) or (iii) TFC (0.26 mg/kg/day) combined into their food pellets or (iv) just food pellets as a negative control. Ciclosporin (70 mg/kg/day) was dissolved in an olive oil/ethanol mixture (9:1 v/v) and administered by oral gavage as a positive control. The mice body weight was monitored throughout the experiment, 1and they were sacrificed 3 days after the last DSS administration. At necropsy, colonic tissues were harvested from the visibly diseased areas, identified by oedema in the DSS model. The colon was carefully dissected, and its length and weight were measured and processed for quantification of inflammatory markers by qPCR as detailed in Section "qPCR quantification of inflammatory markers". Stool consistency was scored using the following scale: 0 = well-formed pellets; 1 = pasty, formed stools; 2 = pasty, semi-formed stools; and 3 = watery diarrhoea.

#### qPCR quantification of inflammatory markers

Colonic tissue samples underwent total messenger ribonucleic acid (mRNA) extraction using a Nucleospin RNA II kit in accordance with the manufacturer’s guidelines (Macherey–Nagel, Düren, Germany). Subsequent reverse transcription was conducted utilising a High-Capacity cDNA Archive Kit, followed by quantitative polymerase chain reaction (qPCR) using SYBR Green (Thermo Fisher Scientific). Primer sequences were designed utilising Primer Express 3 (v3.0.1, Thermo Fisher Scientific). Melting curve analyses were performed for each sample and gene to validate the specificity of amplification. Quantification of target gene expression relied on the comparative cycle threshold (Ct) value, with fold changes in target genes determined utilising the 2^−ΔΔCt^ method.

#### Myeloperoxidase (MPO) activity assay

MPO is present within neutrophils and is released when the immune system is activated thereby acting as a biomarker of inflammation [53]. Mice colons were homogenised in a 0.5% (w/v) hexadecyltrimethylammonium bromide solution in 50 mM PBS. They were subjected to three cycles of freeze-thawing followed by centrifugation at 14,000 × *g* for 15 min at 4 °C. MPO activity was assessed in the resulting clear supernatant by adding dianisidine dihydrochloride at a concentration of 1 mg/mL along with 0.0005% (w/v) hydrogen peroxide (H_2_O_2_). The change in optical density was measured at 450 nm. Human neutrophil MPO served as the standard reference. MPO activity was quantified in terms of units, where one unit of MPO activity corresponded to the degradation of 1.0 µmol of peroxide per minute at 25 °C. To standardise the readings from tissue samples, MPO activity was normalised to the total protein content, which was determined using the DC™ protein assay (Bio-Rad, France).

### Statistical analyses

GraphPad Prism (Version 10.1.1, GraphPad Software LLC, California, USA) was used to plot figures and perform statistical tests. After characterizing the PLGA TFC NPs, the Pearson correlation coefficient was calculated to assess the strength and direction of any correlations between nanoparticle properties and the observed outcomes. Differences between TFC drug release from PLGA NPs were assessed using an unpaired t-test. Differences between study arms for the in vivo assays were assessed using the Mann–Whitney U test. This non-parametric test was chosen to account for any animal losses and to evaluate the differences between two independent variables without assuming a normal distribution of the data. This approach provided a robust and reliable comparison across all treatment groups and time points. In all cases, *p* ≤ 0.05 was considered significant. Significance markers were used accordingly on plots: * *p* ≤ 0.05, ** *p* ≤ 0.01 and *** *p* ≤ 0.001. Unless stated otherwise, bar on charts and points on plots represent mean values and error bars represent standard deviation.

## Results and discussion

### Optimisation of TFC NPs

**Fig. 4:**
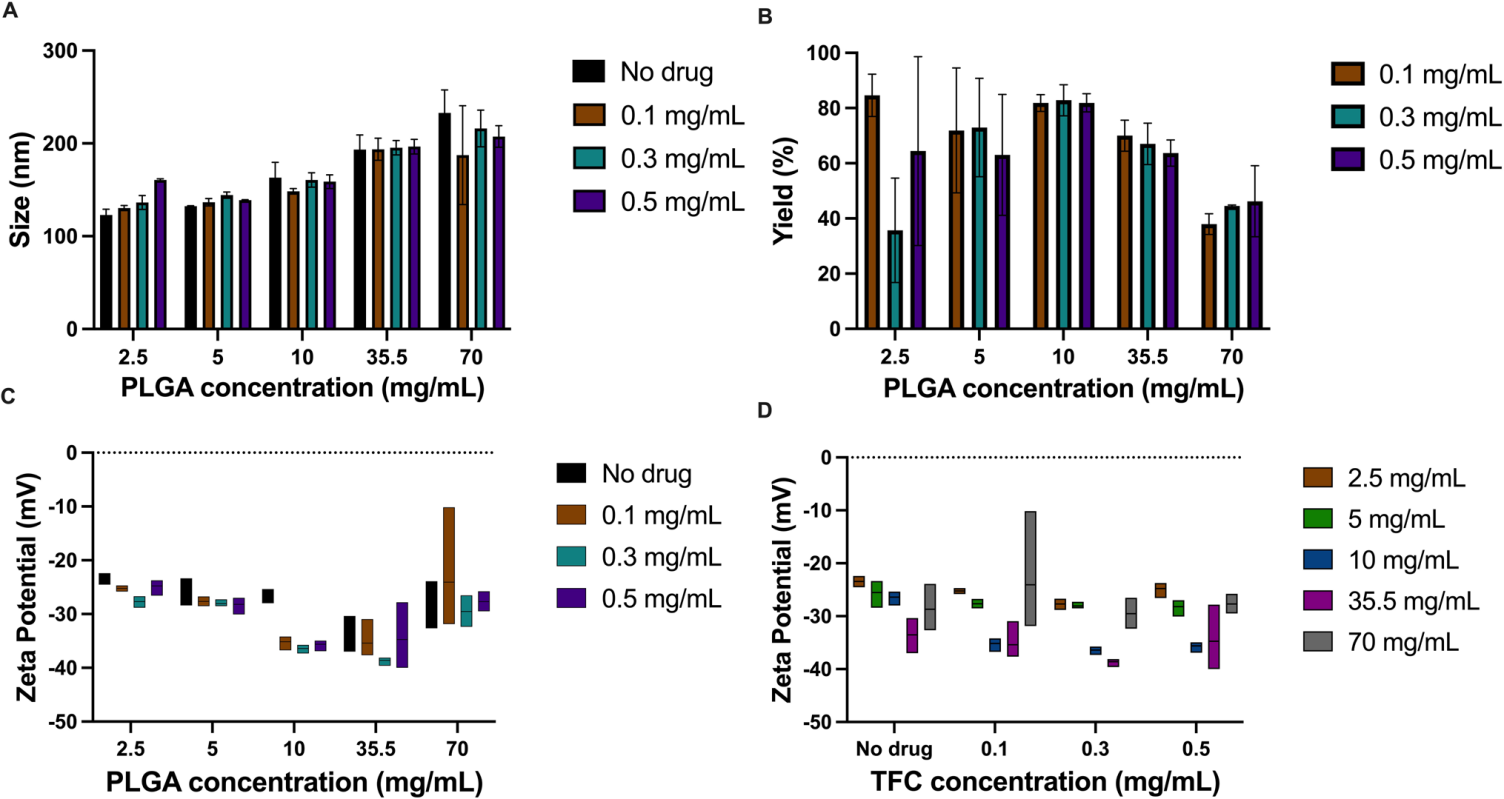
Bar charts illustrating the effects of PLGA and TFC concentrations on (**A**) NP particle size and (**B**) NP product yield calculated using Eq. 3. Box plots illustrating the effects of PLGA and TFC concentrations on (**C**) NP ζ potential based on polymer concentration and (**D**) NP ζ potential based on drug concentration. Each bar within the graphs A-C corresponds to a different tofacitinib concentration (0.1 mg/mL, 0.3 mg/mL and 0.5 mg/mL), and each bar in graph D corresponds to a different PLGA concentration (2,5 mg/mL, 5 mg/mL, 10 mg/mL, 35.5 mg/mL, 70 mg/mL) (*n* = 3). A particle size around 100–150 nm and a ζ potential below -30 mV were desired

To develop a TFC-PLGA nanoparticle formulation capable of targeting the inflamed gut, twenty different combinations TFC PLGA NPs were produced by nanoprecipitation. These combinations varied the concentrations of TFC (0 to 0.5 mg/mL) and PLGA (2.5 to 70 mg/mL). The resulting nanoparticles were characterized for size, charge, drug loading, and drug yield. The enhanced formulation of TFC PLGA NPs was identified through a comprehensive evaluation encompassing size, charge (an indication of physical stability), drug loading, and yield of TFC. The findings revealed that particle size exhibited a direct correlation with PLGA concentrations (*p* < 0.05), while the drug concentration did not exert a significant influence on particle size (*p* = 0.3196) (Fig. 4A and B). Notably, the formulation containing 10 mg/mL PLGA emerged as the most stable, with a ζ potential within the desired range. Interestingly, the drug concentration did not significantly impact particle charge (*p* = 0.1089), contrasting with the pronounced effect observed with varying polymer concentrations (Fig. 4C and D). Particularly, the 10 mg/mL PLGA concentration exhibited the most stable ζ potential (Fig. 4C). However, high variabilities were noted in data associated with higher PLGA concentrations, particularly at 70 mg/mL for particle size and at 35.5 mg/mL and 70 mg/mL for particle stability. This phenomenon is hypothesized to result from enhanced aggregation of NPs within the anti-solvent system, leading to the formation of PLGA clumps during nanoprecipitation. Consequently, this aggregation contributes to the observed reduction in NP yield at higher polymer concentrations. These findings underscore the crucial influence of formulation parameters in influencing the physicochemical properties and stability of TFC PLGA NP. Optimising these parameters is essential to achieve a formulation that balances stability, drug loading, and yield, offering valuable insights for the successful development of targeted NP systems for the treatment of inflammatory conditions of the gut.

**Fig. 5:**
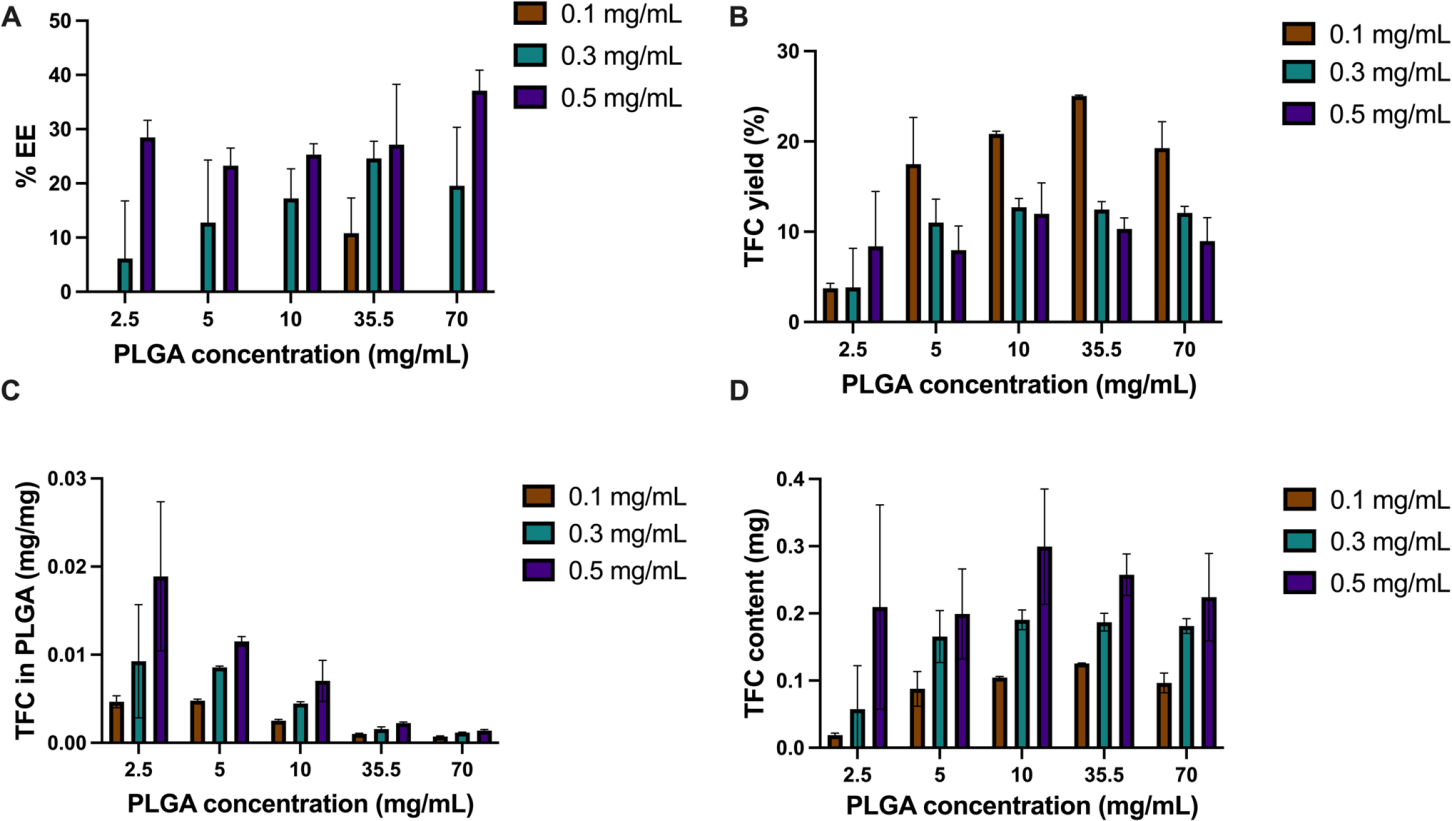
Bar charts illustrating the effects of PLGA and TFC concentrations on (**A**) TFC entrapment efficiency, (**B**) TFC yield, (**C**) drug loading, and (**D**) total TFC content. Each bar within the charts corresponds to a different tofacitinib concentration (0.1 mg/mL, 0.3 mg/mL and 0.5 mg/mL), (*n* = 3)

Furthermore, the impact of TFC concentrations on various parameters critical to NP formulation was investigated. The encapsulation efficiency (EE%) exhibited strong positive correlations with increasing TFC concentrations for formulations PLGA formulations with 2.5 mg/mL (r = 0.950), 5 mg/mL (r = 0.998), 10 mg/mL (r = 0.979), 35.5 mg/mL (r = 0.992) and 70 mg/mL (r = 0.999) (Fig. 5A); suggesting enhanced encapsulation efficiency with increasing drug-to-polymer ratios, which is consistent with previous studies [30, 54]. Interestingly, the current study investigated a formulation with 0.5 mg/mL TFC, revealing an EE% ranging between 20–30%. Notably, this EE% is substantially lower compared to findings reported in the study by Bashir et al., where an EE% of 88% was achieved using a PLGA with a lactide-to-glycolide ratio of 85:15 [30]. This higher lactic acid component renders the polymer more hydrophobic thereby leading to an enhanced affinity of the poorly soluble drugs for this polymer grade [55]. It is noteworthy that some studies did not specify the MW grade of the PLGA utilised. However, it is established in the literature that the entrapment efficiency (EE%) can be influenced by the number of polymer blocks within the PLGA structure. An increased number of polymer blocks extends the diffusional pathways of drugs from the organic phase to the aqueous phase, thus correlating to a reduction in drug loss through diffusion and enhancing drug entrapment efficiency [56, 57]. Herein, in this formulation, a low MW grade of PLGA was employed, characterised by shorter polymer chain lengths. Consequently, this lactide-to-glycolide ratio and low MW PLGA may offer limited capacity for drug encapsulation, thereby explaining the observed lower EE%.

**Fig. 6:**
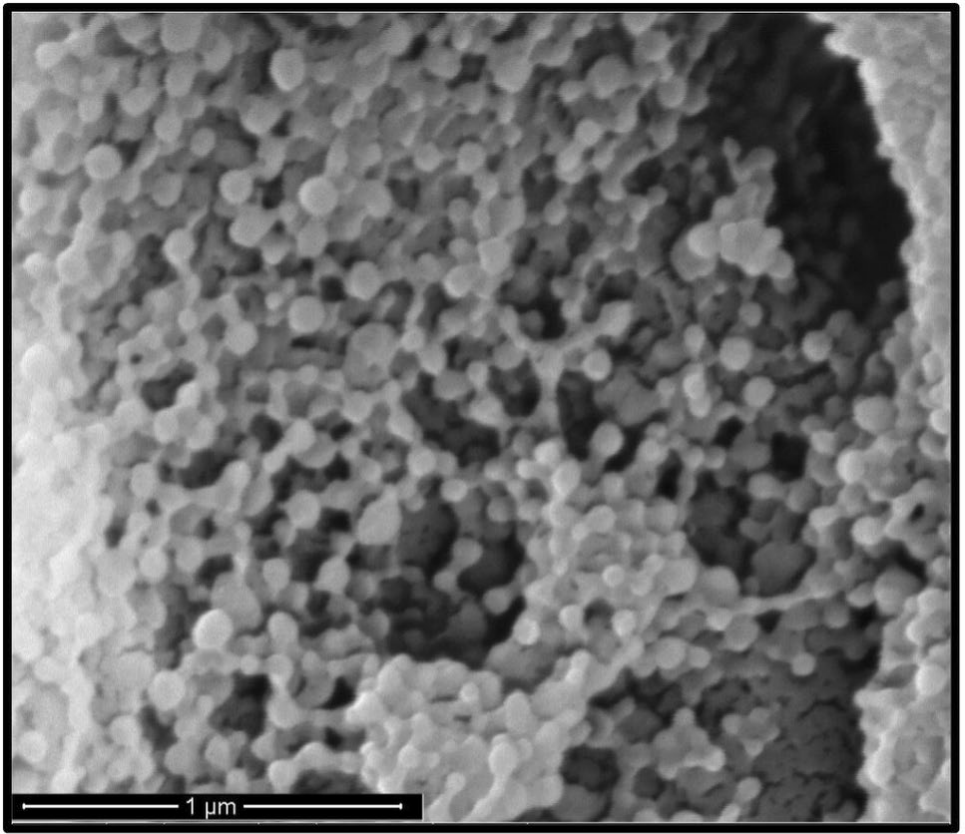
Scanning electron microscopy images of the 10 mg/mL PLGA particles produced by nanoprecipitation following lyophilisation

Conversely, a decrease in TFC yield with increasing TFC concentrations was observed for all formulation except 2.5 mg/mL PLGA (Fig. 5B**)**. The maximum yield using 0.5 mg/mL TFC was observed at the 10 mg/mL PLGA concentration, emphasising the importance of optimising both drug concentration and PLGA concentration for yield maximisation. Notably, Fig. 5C highlights the relationship between TFC concentrations and drug loading, with positive correlations for formulations with 2.5 mg/mL (r = 0.979), 5 mg/mL (r = 0.997), 10 mg/mL (r = 0.997), 35.5 mg/mL (r = 0.999) and 70 mg/mL (r = 0.983) PLGA. Despite the large standard deviations, these results indicate a steady increase in drug loading with higher TFC concentrations, with 0.5 mg/mL TFC concentration exhibiting increased loading efficiency. Considering the expensive nature of TFC, balancing drug loading efficiency with yield considerations is imperative. Figure 5D highlights this point by demonstrating the highest TFC yield at 0.5 mg/mL TFC concentrations in combination with 10 mg/mL PLGA concentrations. In selecting the enhanced TFC PLGA NP formulation, our criteria included particle stability, size below 150 nm, maximal drug loading, and high TFC yield. Through comprehensive evaluation, the formulation comprising 10 mg/mL PLGA and 0.5 mg/mL TFC emerged as the most suitable, meeting all desired criteria for effective TFC PLGA NP production with a particle size of 158.8 ± 7.4 nm and a ζ potential of -35.6 ± 1.1 mV. Figure 6 depicts the scanning electron microscopy image of the selected NP formulation, showing uniform particle size.

### Stability and release profile of TFC from PLGA NPs in simulated gastrointestinal fluids

**Fig. 7:**
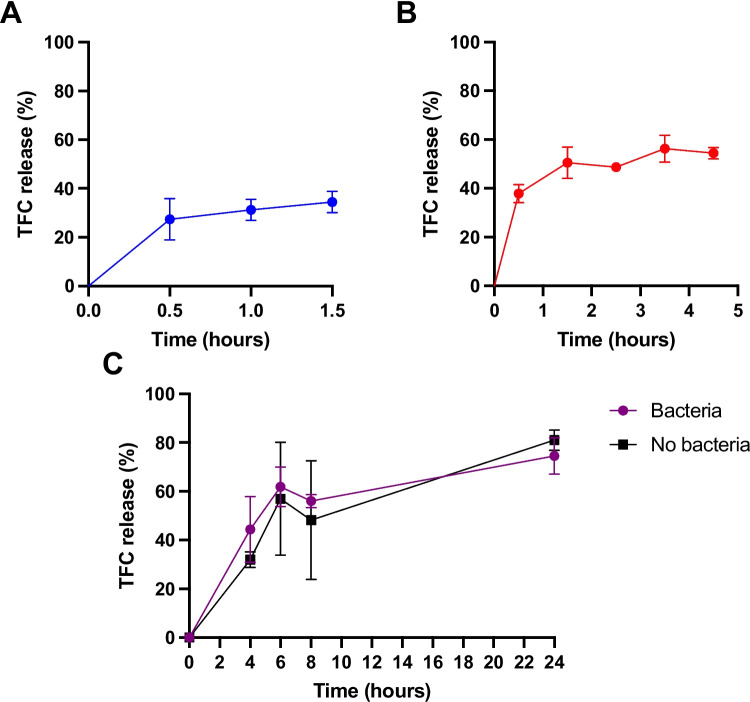
*In* vitro Release Profile of TFC from PLGA Nanoparticles in simulated Gastric Fluid (FaSSGF) (**A**), simulated Intestinal Fluid (FaSSIF) (**B**), and in simulated colonic fluid (BB broth with and without human faecal bacteria) (**C**) (*n* = 3)

In a preliminary study investigating the stability of PLGA in simulated gastric (FaSSGF) and intestinal (FaSSIF) fluids (Supplementary Figure S4), it was found that the low MW grade of PLGA used in this study was mostly stable within the upper GI tract with only minimal degradation in the small intestine (< 5%). This was accordance with previous studies which found higher molecular weight PLGA particles to be relatively stable in the upper GI tract [35, 58]. The release profile of TFC from the PLGA NPs formulated with 10 mg/mL PLGA and 0.5 mg/mL TFC was then assessed across FaSSGF, FaSSIF, and human faecal bacteria fluids (BB Broth). We found an initial burst release of approximately 40% of TFC from the NPs in the 3 compartments (Fig. 7). Subsequently, during gastric (Fig. 7A) and intestinal incubations (Fig. 7B), only approximately 10% and 20% of TFC was released, respectively, following the burst release phase. However, prolonged incubation with human faecal bacteria fluids (Fig. 7C) led to a significant increase in TFC release, with approximately 80% released after 24 h. This release pattern aligns closely with observations from another TFC-PLGA NP study, where a similar release profile was noted after incubation in PBS at pH 7.4 [30]. These results suggest that a substantial portion of the TFC dose would be released in the upper GI tract, thus not achieving complete colon-targeting. This is likely due to the burst release phenomenon, a well-known occurrence with PLGA particles regardless of their route of administration [59–61]. To improve the targeting of the TFC PLGA NPs and ensure the burst release phenomenon occurs at the site of action, a colon-targeted coating can be utilised. Various colon-targeted technologies are available, including pH and time dependent release systems such as Eudragit® RL/RS, which disintegrate upon contact with pH levels greater than 6, thereby releasing their contents in the colonic environment [62]. However, considering that patients with IBD may have more acidic colonic conditions, a dual pH and microbiota-activated system like Phloral® has been developed as a fail-safe mechanism to ensure targeted release in the colon [63]. Future studies may investigate the incorporation of NPs into Phloral®-coated capsules to enhance colonic targeting and improve therapeutic efficacy.

### *In vivo* study: PLGA TFC NP in an acute UC model

**Fig. 8:**
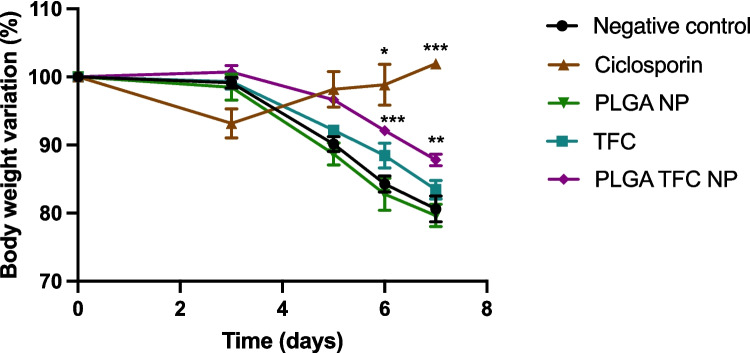
Percentage body weight variations of the DSS C57BL/6 mice following administration of PBS, ciclosporin, PLGA NPs, TFC and PLGA TFC NPs (*n* = 8)

This study proceeded to evaluate the efficacy of the enhanced PLGA TFC NP formulations in vivo*.* Colitis was induced in female mice through the administration of 2.5% DSS in their drinking water. They were, together with the administration of either PLGA NPs, PLGA TFC NPs or TFC combined into their food pellets or just food pellets as a negative control. Ciclosporin, an established treatment for DSS colitis was administered by oral gavage as a positive control [64]. Interestingly, neither TFC alone or PLGA NPs alone showed a significant effect on weight variation although a significant increase in weight gain was observed with the same amount of TFC when delivered within PLGA NPs (*P* < 0.005) (Fig. 8). This finding highlights the potential of the nano-targeting approach employed in this study, emphasising the enhanced efficacy of TFC when encapsulated within PLGA NPs. This nano-approach was also found to be promising in a study by Li et al. where a TFC loaded albumin nanomedicine administered intravenously was capable of targeting the colon of colitis mice and reducing pro-inflammatory cytokines [65]. Surprisingly, the administration of PLGA NPs did not result in a change in body weight, contrasting with previous in vitro colitis model findings [31]. This may be due to the smaller size of the NPs used in this study, which is expected to degrade into lactic acid more readily than microparticles, potentially causing excessive acidification of the colonic environment, which may adversely affect gut microbiota (see Figure S4).

**Fig. 9:**
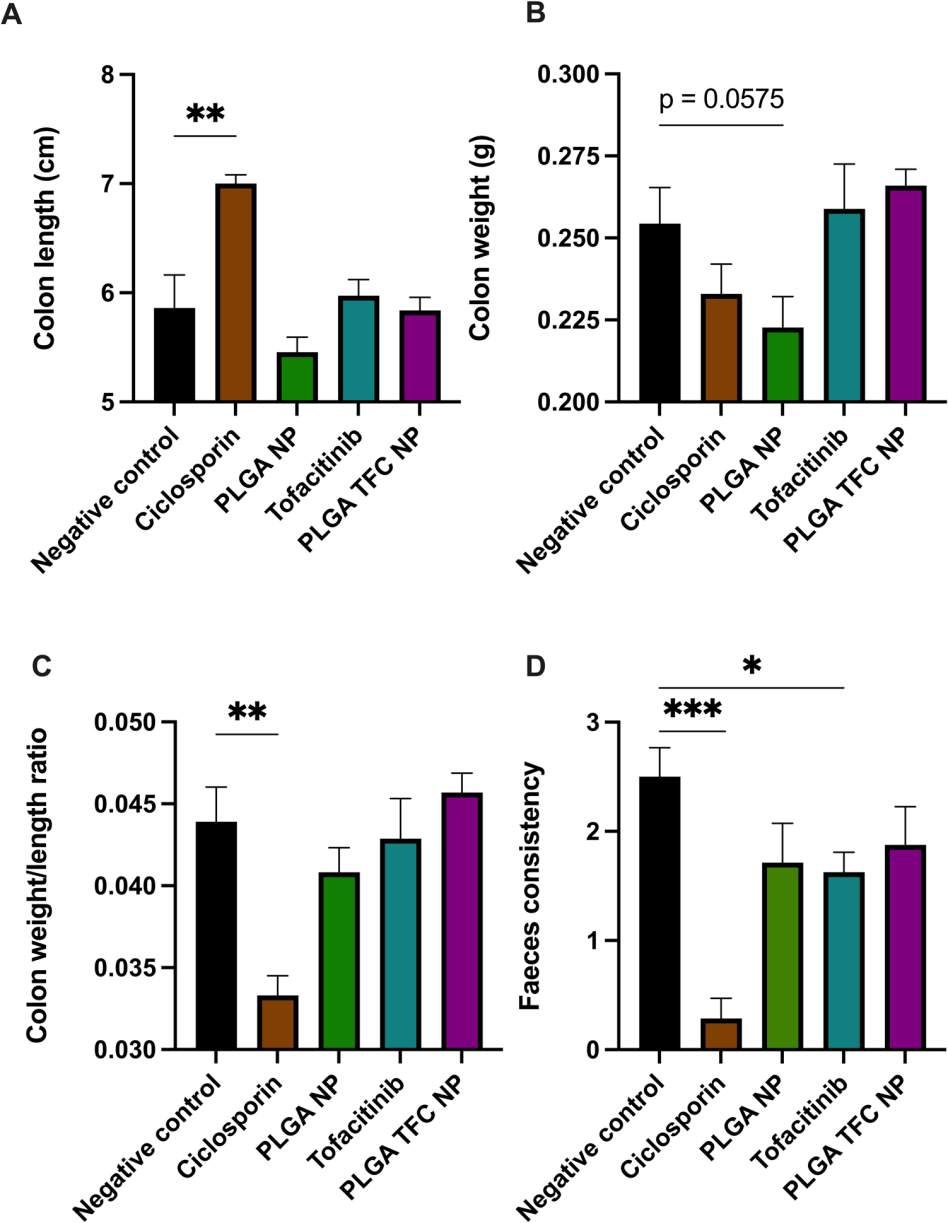
Bar charts showing the effect of PLGA NPs, TFC or PLGA TFC NPs administration on colon length (**A**) and colon weight (**B**) the colon weight to length ratio (**C**) and on the faeces consistency (**D**) of a DSS colitis mice (*n* = 8). Significance markers were used according to the Mann–Whitney U test: * for *p* ≤ 0.05, ** for *p* ≤ 0.01, and *** for *p* ≤ 0.001

Macroscopic and clinical analysis revealed distinct outcomes across experimental groups. Treatment with the positive control, ciclosporin, exhibited significant preventive effects on colitis progression, illustrated by a notable prevention of colon shortening (*p* < 0.005) (Fig. 9A**)** and a reduced scoring of faeces consistency (*p* < 0.001) indicating the presence of normal to softer stools compared to loose stools for the DSS control (Fig. 9D). Additionally, Fig. 9C demonstrates a lower colon weight/length ratio in the ciclosporin-treated group compared to the control (*p* < 0.005), further supporting the protective effects of this treatment. Treatment with TFC alone resulted in reductions only in faeces consistency (*p* < 0.05), although surprisingly without significant effects observed on colon weight, length, or the weight/length ratio. No significant differences were observed in macroscopic and clinical scoring between the DSS control group and mice treated with PLGA NPs or PLGA TFC NPs, although a trend toward an improvement in the faeces consistency score in both groups was observed. The efficacy and administration of TFC in conjunction with PLGA NPs reveal several pertinent considerations. Notably, variations in TFC effectiveness are likely influenced by differences in food intake among experimental animals, given that TFC was administered with food rather than through oral gavage due to lyophilised particle aggregation occurring specifically under the mechanical stress exerted during passage through the oral gavage tubes [66]. Additionally, the the impact of particle size and the severity of colitis were also considered. Unfortunately, the highly negative ζ potential on the surface of the NPs was insufficient on its own to tackle particle aggregation. To address this issue, we propose the further exploration of chitosan coating on NPs [67] or the use of a cryoprotectant prior to lyophilisation [68] as additional measures to facilitate oral gavage administration and mitigate aggregation concerns, thus ensuring more consistent dosing regimens.

**Fig. 10:**
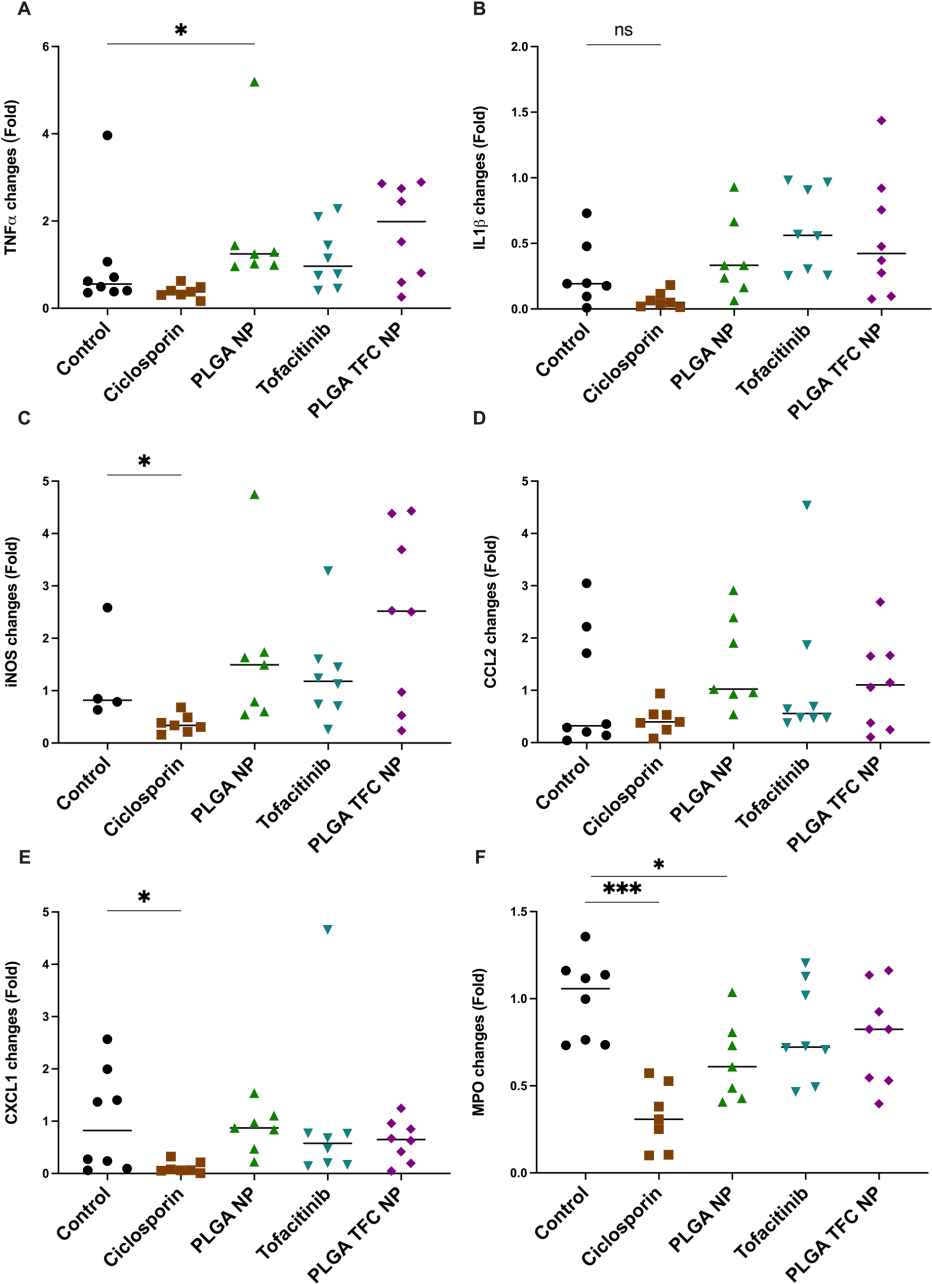
Scatter plots of qPCR analyses of colonic the inflammatory markers (**A**) *Tnf-α*, (**B**) *Il-1b*, (**C**) *inos*, (**D**) *Ccl2*, (**E**) *Cxcl1* and (**F**) of MPO activity (*n* = 8). Significance markers were used according to the Mann–Whitney U test: * for *p* ≤ 0.05, ** for *p* ≤ 0.01, and *** for *p* ≤ 0.001

Analysis of colonic inflammatory markers via qPCR revealed distinct patterns of gene expression across experimental groups (Fig. 10**)**. Treatment with the positive control, ciclosporin, resulted in significant reductions in the expression levels of the inflammatory markers *Tnf-α*, *inos, Cxcl1* (*p* < 0.05) and of MPO activity (*P* < 0.001). These findings confirm the ameliorating potential of this colitis mouse model. In contrast, no significant changes in the expression levels of inflammatory markers were observed with TFC alone or PLGA TFC NPs. PLGA NPs alone had a significant effect on the MPO activity (*p* < 0.05) but significantly increased the production of *Tnf-α* (*p* < 0.05). A trend toward an improvement in term of MPO activity was nevertheless observed for the three test groups.

These results suggest that TFC administered at a dosage of 0.26 mg/kg/day does not exert a therapeutic effect on colonic inflammatory markers in this experimental model. Overall, these findings highlight that the drug dose administered in this study was insufficient to significantly modulate inflammatory markers. In the context of this study, the challenge of balancing dose effects between PLGA and TFC emerges, compounded by formulation constraints. This study faced the challenge of balancing dose effects between PLGA and TFC, which was further complicated by formulation constraints. The TFC dose used corresponded to the allometric conversion of the human TFC dose and the human in vitro PLGA dose (2.50 g) as reported by McCoubrey et al., using the enhanced PLGA TFC nanoparticle formulation [31]. This dose was justified by previous findings in male Lewis rats, where colon-targeted delivery of TFC (10 mg/kg) resulted in increased colonic tissue exposure to the drug compared to the immediate release formulation [16]. Additionally, the shorter GI transit time of 1 to 6 h in mice [69] compared to humans, prompted the design of this study to use a lower dose based on targeted delivery to the colon as most of the drug would be delivered in the colonic environment.

Another limitation of this study was the administration of TFC based on an allometric conversion, which resulted in a lower dose compared to those used in previous in vivo studies. Specifically, other studies have utilised doses of 10 mg/kg or 30 mg/kg in C57BL/6 acute DSS mouse models, suggesting that the dose used in our study may have been insufficient [70, 71]. These high doses were likely required due to its highly metabolised characteristic which indicates that allometric conversion was not the most suitable approach. Interestingly, in a previous study, TFC at 10 mg/kg showed only improvement in weight loss when given in an acute rescue DSS model but no significant improvement in disease activity index (DAI), histopathology, or inflammatory markers. However, at the higher 30 mg/kg dose, the improvement in weight loss is accompanied by improvements in DAI, histopathology, and inflammatory markers. Similarly, in the recent study by Seal et al. using a chronic DSS model of C57BL/6 mice with three cycles of DSS, they found only histopathology effects but no effects on inflammatory markers for TFC when started after the first or second cycle of DSS [72]. However, when started only after a second or third cycle of DSS, TFC was found to have positive effects on histopathology and inflammatory markers, thereby indicating that late administration of TFC in chronic DSS mouse models may be the most suitable in vivo assay.

Altogether, these results may suggest that the PLGA TFC NPs are superior to TFC alone since the dose used for PLGA TFC NPs was too low to exert an effect on histopathology and inflammatory markers, but still showed an effect on weight loss prevention, whereas TFC alone had no significant effect across all assays. To navigate this challenge, we suggest conducting separate dose-finding assays for PLGA and PLGA TFC NPs in acute rescue or chronic DSS mice models to enhance our understanding of their dose–response relationships. In summary, the current results show the potential superiority of NP delivery over TFC alone and emphasise the importance of optimising dosing strategies in future studies to maximise therapeutic outcomes.

## Conclusion

In this study, the production of TFC PLGA NPs was optimised, balancing particle size, stability, drug loading and drug yield. We then incubated the selected formulation in gastric, intestinal, and colonic microbiota fluids to assess drug release. The NPs were found to release around 40% of their TFC content by burst release, followed by gradual release, with up to 80% TFC release after 24 h in the colonic microbiota environment. These results indicate that the TFC PLGA NPs enable release of TFC in the GI tract and that they are promising for IBD targeting if encapsulated with a colon targeted enteric coating. The findings demonstrate the potential of PLGA TFC NPs in mitigating weight loss in an IBD mouse model while highlighting the necessity for further dose-finding assays to fully understand their therapeutic efficacy. Overall, the superior performance of PLGA TFC NPs compared to TFC alone suggests the promising role of NP delivery systems in enhancing drug efficacy, marking a significant step towards reducing dosage and associated side effects in IBD treatment. Future work may involve increasing the dose of TFC in in-vivo studies and administering the nanoparticles in capsules with colon-targeted coatings to enhance dose precision, potentially improving therapeutic outcomes.

## Supplementary Information

Below is the link to the electronic supplementary material.

Supplementary file1 (DOCX 76.3 KB)

## Data Availability

All data generated or analysed during this study are included in this published article and its supplementary information file.
