# Supplementary material for: Dual action tofacitinib-loaded PLGA nanoparticles alleviate colitis in an IBD mouse model
Source: Drug Deliv Transl Res. 2024 Nov 11;15(7):2372–89. doi: 10.1007/s13346-024-01736-1 (PMC12137538; doi:10.1007/s13346-024-01736-1)

Supplementary Materials for:

**Dual action tofacitinib-loaded PLGA nanoparticles alleviate colitis in an IBD mouse model.**

Nidhi Seegobin^1^, Laura E. McCoubrey^1^, Cécile Vignal^2^, Christophe Waxin^2^, Youssef Abdalla^1^, Yue Fang^1^, Atheer Awad^1,3^, Sudaxshina Murdan^1^, Abdul W. Basit^1^

^1^ Department of Pharmaceutics, UCL School of Pharmacy, University College London, 29-39 Brunswick Square, London, WC1N 1AX, United Kingdom.

^2^ Univ. Lille, Inserm, CHU Lille, UMR1286 - INFINITE - Institute for Translational Research in Inflammation, F-59000 Lille, France

^3^ Department of Clinical, Pharmaceutical and Biological Sciences, University of Hertfordshire, College Lane, Hatfield, AL10 9AB, United Kingdom.

*Correspondence: a.basit@ucl.ac.uk

Supplementary Table S1. Formulation combinations for the optimisation of TFC PLGA NPs using a full factorial Design of Experiment and their associated physical characterisation results (n=3).

| Formulation | PLGA concentration  (mg/mL) | TFC concentration  (mg/mL) | Particle size (nm) | Zeta Potential (mV) | TFC content (mg) |
| --- | --- | --- | --- | --- | --- |
| 2.5-0 | 2.5 | - | 122.8 ± 6.3 | -23.4 ± 1.5 | - |
| 2.5-0.1 | 2.5 | 0.1 | 130.4 ± 2.8 | -25.2 ± 0.6 | 0.02 ± 0.00 |
| 2.5-0.3 | 2.5 | 0.3 | 136.5 ± 7.5 | -27.7 ± 1.1 | 0.06 ± 0.06 |
| 2.5-0.5 | 2.5 | 0.5 | 160.5 ± 1.3 | -24.8 ± 1.6 | 0.21 ± 0.15 |
| 5-0 | 5 | - | 132.4 ± 0.5 | -25.5 ± 2.6 | - |
| 5-0.1 | 5 | 0.1 | 136.7 ± 3.9 | -27.7 ± 0.9 | 0.09 ± 0.03 |
| 5-0.3 | 5 | 0.5 | 144.3 ± 3.4 | -28.1 ± 0.7 | 0.17 ± 0.04 |
| 5-0.5 | 5 | 0.3 | 138.9 ± 0.5 | -28.2 ± 1.6 | 0.20 ± 0.07 |
| 10-0 | 10 | - | 163.1 ± 16.5 | -26.4 ± 1.4 | - |
| 10-0.1 | 10 | 0.1 | 148.4 ± 3.0 | -35.1 ± 1.4 | 0.10 ± 0.00 |
| 10-0.3 | 10 | 0.3 | 160.7 ± 7.9 | -36.4 ± 0.8 | 0.19 ± 0.01 |
| 10-0.5 | 10 | 0.5 | 158.8 ± 7.4 | -35.6 ± 1.1 | 0.30 ± 0.09 |
| 35.5-0 | 35.5 | - | 193.4 ± 15.8 | -33.5 ± 3.3 | - |
| 35.5-0.1 | 35.5 | 0.1 | 193.7 ± 11.9 | -35.4 ± 3.8 | 0.13 ± 0.00 |
| 35.5-0.3 | 35.5 | 0.3 | 195.4 ± 7.7 | -38.6 ± 0.8 | 0.19 ± 0.01 |
| 35.5-0.5 | 35.5 | 0.5 | 196.5 ± 7.8 | -34.7 ± 6.2 | 0.26 ± 0.03 |
| 70 - 0 | 70 | - | 232.8 ± 24.7 | -28.7 ± 4.4 | - |
| 70-0.1 | 70 | 0.1 | 187.3 ± 53.2 | -24.1 ± 12.1 | 0.10 ± 0.01 |
| 70-0.3 | 70 | 0.3 | 216.1 ± 19.7 | -29.5 ± 2.9 | 0.18 ± 0.01 |
| 70-0.5 | 70 | 0.5 | 207.5 ± 11.6 | -27.7 ± 1.9 | 0.22 ± 0.06 |

Supplementary Table S2. The composition of the simulated fluids used to predict PLGA stability in the proximal GI tract.

| **FaSSGF** | | |
| --- | --- | --- |
| **Component** | **Concentration** | **Function** |
| Sodium taurocholate | 80 µM | Bile salt: wetting and solubilisation agent |
| Lecithin | 20 µM | Phospholipid: wetting and solubilisation agent |
| Pepsin | 0.1 mg/mL | Enzyme: biorelevant metabolism |
| Sodium chloride | 34.2 mM | Salt: adjustment of biorelevant osmolality |
| Lipase (from porcine pancreas) | 100 µg/mL | Enzyme: biorelevant metabolism |
| Hydrochloric acid | Adjusted to pH 1.60 | Acid: biorelevant pH adjustment |
| **FaSSIF** | | |
| **Component** | **Concentration** | **Function** |
| Sodium taurocholate | 3 mM | Bile salt: wetting and solubilisation agent |
| Lecithin | 0.75 mM | Phospholipid: wetting and solubilisation agent |
| Potassium dihydrogen phosphate | 28.65 mM | Salt: buffering agent |
| Sodium chloride | 105.85 mM | Salt: adjustment of biorelevant osmolality |
| Sodium hydroxide | 10.5 mM | Base: biorelevant pH adjustment |
| Lipase (from porcine pancreas) | 20 mg/mL | Enzyme: biorelevant metabolism |
| Hydrochloric acid/additional sodium hydroxide | Adjusted to pH 6.50 | Acid/Base: biorelevant pH adjustment |
| **Blank FaSSIF** | | |
| **Component** | **Concentration** | **Function** |
| Potassium dihydrogen phosphate | 28.65 mM | Salt: buffering agent |
| Sodium chloride | 105.85 mM | Salt: adjustment of biorelevant osmolality |
| Sodium hydroxide | 10.5 mM | Base: biorelevant pH adjustment |
| Hydrochloric acid/additional sodium hydroxide | Adjusted to pH 6.50 | Acid/Base: biorelevant pH adjustment |

### Supplementary methods S3. Collection and culture of human faecal microbiota

Faecal samples were collected from six healthy volunteers (3 males, 3 females) with diverse ethnic backgrounds, all of whom had abstained from oral antibiotics for a minimum of 12 months. Ethical clearance for human faecal sample collection was secured from the UCL Biobank Ethical Review Committee at Royal Free London NHS Foundation Trust (reference no. NC2017.010), as granted to Intract Pharma Ltd. To ensure optimal culturing conditions, an anaerobic chamber (Electrotek 500TG workstation, Electrotek, West Yorkshire, UK) was utilized. Faecal slurries were prepared by homogenizing faecal samples with basal media at a 1:3 ratio, resulting in a 25% w/w concentration. The composition of the basal media followed a prior study [54]. After sieving through a 250 µm pore size SefarNitex TM sieve, 0.50 mL of faecal slurry was inoculated into 100 mL sterilized Bryant and Burkey broth and incubated anaerobically for 24 hours to allow the faecal microbiota to establish. Subsequently, 1.0 mL of the resulting culture was transferred to a second 100 mL sterile Bryant and Burkey broth and incubated for an additional 24 hours under anaerobic conditions to ensure stable and abundant samples. The final culture was mixed with 30% glycerol in ¼ Ringer’s solution to yield a 15% v/v glycerol stock suspension, which was then aliquoted and frozen at -80 °C for each donor. The addition of ¼ Ringer’s solution and glycerol served to maintain microbial viability during freezing and storage. Each aliquot was utilized only once to prevent multiple freeze-thaw cycles and preserve microbial viability [55].

Supplementary Table S3. Colony Forming Units (CFU) per Millilitre of Fluid in Faecal Microbiota Aliquots from Six Healthy Human Donors. Results are representative of one replicate.

| Donor | CFU/mL |
| --- | --- |
| Male 1 | 1.12E+09 |
| Male 2 | 8.60E+08 |
| Male 3 | 8.60E+08 |
| Female 1 | 8.80E+08 |
| Female 2 | 1.02E+09 |
| Female 3 | 6.80E+08 |

Supplementary Figure S4. The stabilities of PLGA particles in simulated gastric and small intestinal fluids, as measured by a change in pH, with FaSSGF + lipase (100 µg/mL) (A), FaSSIF + lipase (20 mg/mL) (B), and blank FaSSIF (C) in the presence of PLGA A, PLGA B, and PLA nanoparticles. Measurements were conducted in triplicate.


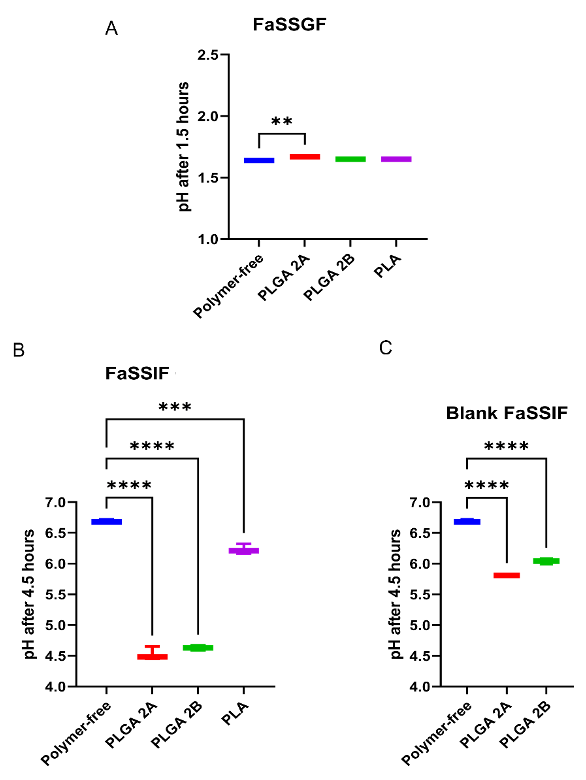

Supplement: Supplementary file 1 — Supplementary file1 (DOCX 76.3 KB) [file 13346_2024_1736_MOESM1_ESM.docx]
